# Interaction of tRNA with MEK2 in pancreatic cancer cells

**DOI:** 10.1038/srep28260

**Published:** 2016-06-15

**Authors:** Xiaoyun Wang, Christina R. Chow, Kazumi Ebine, Jiyoung Lee, Marsha R. Rosner, Tao Pan, Hidayatullah G. Munshi

**Affiliations:** 1 Department of Biochemistry and Molecular Biology, University of Chicago, Chicago, IL 60637, USA; 2 Department of Medicine and the Robert H. Lurie Comprehensive Cancer Center, Northwestern University, Chicago, IL 60611, USA; 3 Ben May Department for Cancer Research, University of Chicago, Chicago, IL 60637, USA

## Abstract

Although the translational function of tRNA has long been established, extra translational functions of tRNA are still being discovered. We previously developed a computational method to systematically predict new tRNA-protein complexes and experimentally validated six candidate proteins, including the mitogen-activated protein kinase kinase 2 (MEK2), that interact with tRNA in HEK293T cells. However, consequences of the interaction between tRNA and these proteins remain to be elucidated. Here we tested the consequence of the interaction between tRNA and MEK2 in pancreatic cancer cell lines. We also generated disease and drug resistance-derived MEK2 mutants (Q60P, P128Q, S154F, E207K) to evaluate the function of the tRNA-MEK2 interaction. Our results demonstrate that tRNA interacts with the wild-type and mutant MEK2 in pancreatic cancer cells; furthermore, the MEK2 inhibitor U0126 significantly reduces the tRNA-MEK2 interaction. In addition, tRNA affects the catalytic activity of the wild type and mutant MEK2 proteins in different ways. Overall, our findings demonstrate the interaction of tRNA with MEK2 in pancreatic cancer cells and suggest that tRNA may impact MEK2 activity in cancer cells.

Although the translational function of tRNA has long been established, extra translational functions of tRNA are still being discovered. Previously known extra translational functions of tRNA were identified in a case-by-case basis^1^,^2^,^3^. To systematically identify new tRNA-protein complexes that may perform extra-translational function, we previously developed a computational method to predict new tRNA-protein complexes and identified 37 mammalian protein candidates that could potentially bind tRNA^4^. Most were enzymes involved in cellular processes unrelated to translation and were not known to interact with nucleic acids before. We experimentally confirmed six candidate proteins for tRNA binding in HEK293T cells using anti-EF-1α as positive and anti-GFP and IgG as negative controls^4^. They include the metabolic enzyme phosphoenolpyruvate carboxykinase, protein modification enzyme farnesyltransferase, a GTPase involved in membrane trafficking SAR1a, the euchromatic histone methyltransferase 1, glutathione synthetases, and mitogen-activated protein kinase kinase 2 (MEK2). However, biological consequences of these tRNA-protein interactions remain to be elucidated.

The discovery of many tRNA-binding proteins suggests a widespread, non-canonical role for tRNA-protein interactions in cellular communications between translation and other cellular processes. In this model, when translation activity is high, most tRNAs are used by the ribosome and only a small amount of tRNA is available to interact with other proteins. When translation activity is low, more tRNA becomes available to interact with other proteins, which may result in up- or down-regulation of other cellular processes. In this current work using pancreatic cancer cell lines, we evaluated the effects of the interaction between tRNA and MEK2 which is one of the six proteins that we experimentally validated to interact with tRNA in our previous work^4^. The original finding of tRNA-MEK2 interaction was performed in HEK293T cells. We used UV crosslinking-immunoprecipitation followed by tRNA microarray (CLIP-Chip), a widely applied technique to investigate RNA-protein interactions^5^,^6^. To determine the function of the tRNA-MEK2 interaction, we evaluated the effects of tRNA on the catalytic activity of the wild-type MEK2 and several MEK2 mutants that were shown previously to cause developmental defects (P128Q) or associate with resistance to MEK inhibitors (Q60P, S154F, E207K)^7^,^8^,^9^. Our results demonstrate that tRNA interacts with MEK2 and its mutants in pancreatic cancer cells and that the MEK-specific inhibitor U0126 reduces the tRNA-MEK2 interaction in cells. Biochemical assays show that human tRNA reduces the catalytic activities of the wild type protein, but can increase the activity of certain mutant MEK2 proteins, especially the P128Q mutant. Overall, our findings demonstrate the interaction of tRNA with MEK2 in pancreatic cancer cells and tRNA affecting the catalytic activity of MEK2 proteins. tRNA may modulate MEK2 function to regulate cellular behavior.

## Results and Discussion

### tRNA and MEK2 interaction in pancreatic cancer cells and in a non-tumorigenic cell line

**Figure 1:**
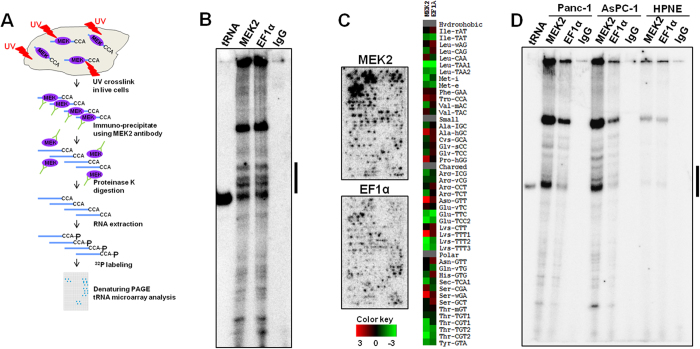
tRNA and MEK2 interaction in pancreatic cancer cells and in a non-tumorigenic cell line. (**A**) Flow chart of CLIP-Chip. Cells growing on tissue culture plastic were exposed to UV to crosslink RNA and proteins in live cells and then processed for CLIP-Chip using specific antibody against MEK2. (**B**) Denaturing PAGE analysis was performed to evaluate MEK2-tRNA interaction in CD18 pancreatic cancer cells. EF1α antibody was used as a positive control, and IgG as a negative control. The indicated tRNA bands were cut out for tRNA microarray. (**C**) tRNA microarrays for semi-quantitative analysis of CLIP-Chip array results, and representative arrays are shown. Anticodon specific tRNA signals are calculated and expressed as log_2_(X), where X indicates values relative to the median value of each array. Relative abundance of specific tRNAs are shown as heat map and are indicated with colors ranging from green (low) to red (high). (**D**) PAGE analysis was performed to evaluate crosslinked tRNAs to MEK2 and EF1α in Panc-1 and AsPC-1 pancreatic cancer cells, and in the non-tumorigenic pancreatic cancer cell line HPNE.

Since the original finding demonstrating tRNA-MEK2 interaction was performed in HEK293T cells, we evaluated whether tRNA and MEK2 also interacts in pancreatic cancer cells. CD18 pancreatic cancer cells growing on tissue culture plastic were exposed to UV to crosslink RNA with proteins in live cells and then processed for CLIP-Chip using the antibody against MEK2 (Fig. 1A). Antibody against the translational elongation factor EF1α was used as a positive control, and IgG was used as a negative control. Denaturing gel electrophoresis of ^32^P-labeled and MEK2-crosslinked RNA showed strong bands corresponding to the full-length tRNAs that were also present in the positive control (Fig. 1B). tRNA microarray analysis^4^,^10^ demonstrated tRNA binding for both MEK2 and EF1α, but with some quantitative differences in the crosslinked tRNA species, suggesting that some tRNAs preferentially interact with MEK2 in CD18 cells (Fig. 1C) when referred to the relative tRNA abundance in different pancreatic cell lines (Fig. S1). We also evaluated to what extent tRNA and MEK2 interact in other pancreatic cell lines (Fig. 1D). tRNAs also interacted with MEK2 in the malignant AsPC1 and Panc1 cells and in the immortalized HPNE cell. MEK2 interaction with specific tRNAs is selective as indicated by similar MEK2 and EF1α expression levels in these pancreatic cell lines (Fig. S2).

### The MEK inhibitor U0126 decreases MEK2-tRNA interaction in pancreatic cancer cells

**Figure 2:**
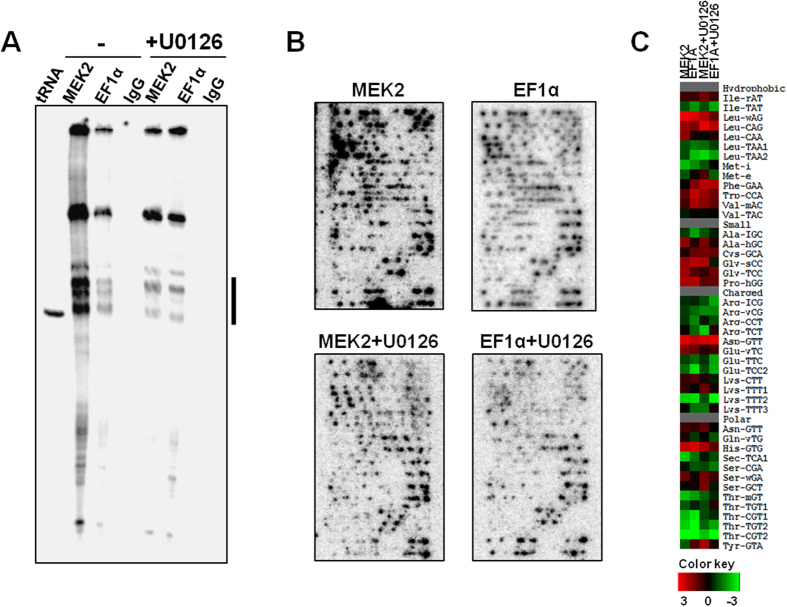
The MEK inhibitor U0126 decreases MEK2-tRNA interaction in pancreatic cancer cells. (**A**) CD18 pancreatic cancer cells growing on tissue culture plastic were treated with DMSO (vehicle control) or MEK1/2 inhibitor U0126 for 4 hours, exposed to UV to crosslink RNA and protein in live cells and then processed for CLIP-Chip using MEK2 antibody, EF1α antibody (positive control) or IgG antibody (negative control). Denaturing PAGE and tRNA microarray analysis demonstrating MEK2-tRNA interactions, with the indicated tRNA bands cut out for array analysis. (**B**) Semi-quantitative tRNA microarray analysis of tRNA species bound to MEK2/EF1α in CD18 pancreatic cancer cells. (**C**) Anticodon specific tRNA signals are calculated and expressed as log_2_(X), where X indicates values relative to samples treated with DMSO (vehicle control). Relative abundance of specific tRNAs are shown as heat map and are indicated with colors ranging from green (low) to red (high).

We next evaluated whether blocking the MEK2 activity affected the interaction between MEK2 and tRNA in CD18 cells. The MEK2 activity was blocked using the well-established MEK1/2 inhibitor U0126^11^. CD18 cells were treated with U0126 for 4 hours, exposed to UV for RNA-protein crosslinking and then processed for CLIP-Chip assay. When normalized to the amount of tRNAs crosslinked to EF1α, tRNA crosslinked to MEK2 were markedly reduced in the presence of U0126 (Fig. 2A). This reduction is validated by tRNA microarray analysis (Fig. 2B), and the heat map shows preferred binding tRNA species from CLIP-Chip results (Fig. 2C). The difference between the crosslinked tRNAs to EF-1α is substantially smaller than those to MEK2. These results indicate that tRNA preferably interacts with active MEK2 in cells; alternatively, U0126 and tRNA binding sites overlap in the MEK2 protein.

### Interaction of tRNA with MEK2 mutants

**Figure 3:**
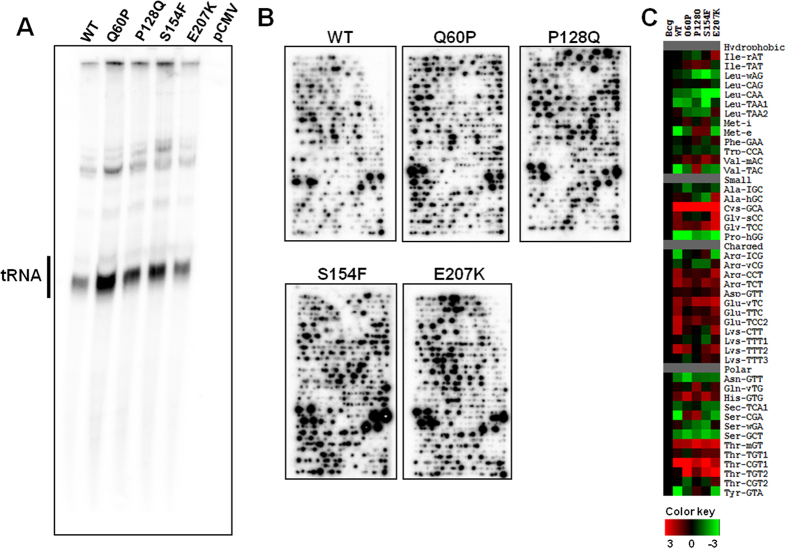
Interaction of tRNA with disease-associated MEK2 mutants. (**A**,**B**) HEK293T cells transfected with Flag-tagged, wild type MEK2 or MEK2 mutants previously associated with developmental defects, cancer development or resistance to targeted therapy. The cells were then exposed to UV to crosslink RNA and protein in live cells and processed for CLIP-Chip using specific antibody against MEK2. PAGE analysis was performed to evaluate MEK2-tRNA interactions. The indicated tRNA bands were cut out for microarray analysis and for semi-quantitative analysis of CLIP-Chip array results. (**C**) Anticodon specific tRNA signals are calculated and expressed as log_2_(X), where X indicates values relative to the background (Bcg) signal present in HEK293T cells (data from our previous work^4^). Relative abundance of specific tRNAs are shown as heat map and are indicated with colors ranging from green (low) to red (high).

To further evaluate the interaction between MEK2 and tRNA in cells, we selected MEK2 mutants that were identified as cause of developmental defects or were previously shown to be associated with chemo-therapy resistance to MEK inhibitors in melanoma. The P128Q MEK2 mutation, which is known to increase the activity of MEK2, causes cardiofaciocutaneous (CFC) syndrome^8^. The Q60P, S154F, and E207K mutations were identified in melanoma patients developing resistance to MEK inhibitors^7^,^9^. CLIP-Chip analysis demonstrated that tRNAs interact with the wild-type MEK2 and the various MEK2 mutants in HEK293T cells (Figs 3A and S1). Western blot showed that different MEK2 plasmids have similar expression level after transfection (Fig. S3), while the tRNA microarray analysis demonstrated increased binding of tRNAs to MEK2 mutants compared to the wild-type MEK2, the binding selectivity of tRNA species is similar for the wild-type and the MEK2 mutants (Fig. 3B,C). These results indicate that these pathologically relevant MEK2 mutations can increase binding of tRNAs to MEK2, but without significantly affecting the composition of the tRNAs that bind to MEK2.

### Effects of tRNA on MEK2 activity

**Figure 4:**
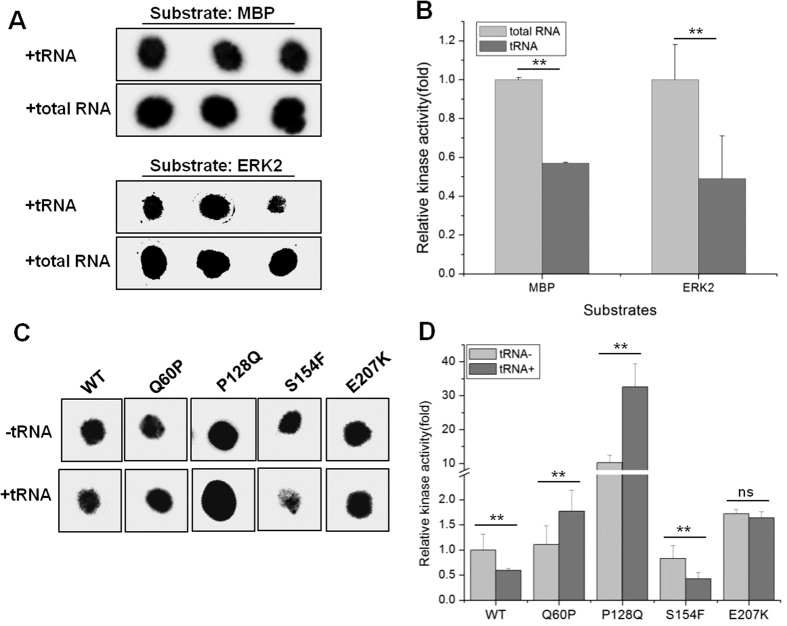
Effect on catalytic activity of the different MEK2 mutants by tRNA. (**A**) *In vitro* kinase dot blot assays using wild-type MEK2 were performed with MBP protein or ERK2 as substrate, in the presence of human tRNA or human total RNA (0.2 μg/μl). Kinase assay without MEK2 protein was used as background control. (**B**) MEK2 kinase activity was quantified, and normalized to the activity of wild-type MEK2 in the presence of human total RNA. Kinase assays were performed in triplicate, and error bars represent standard deviations from these replicates. **indicates P < 0.05. (**C**) Wild-type MEK2 or MEK2 mutants (Q60P, P128Q, S154F and E207K) were used in the kinase dot blot assays using MBP peptide as substrate, in the presence or absence of human tRNA. (**D**) MEK2 kinase activity was quantified, and normalized relative to the activity of wild-type MEK2 in the absence of tRNA. Kinase assays were performed in quadruplicate or triplicate, and error bars represent standard deviations from these replicates. Representative dots of kinase assays are shown. **indicates P < 0.05 relative to kinase activity without RNA, ns indicates no significant difference.

We next evaluated the effects of the tRNA-MEK2 interaction on the catalytic activity of the different MEK2 mutants using *in vitro* kinase dot blot assays^12^,^13^,^14^ and purified, Flag-tagged MEK2 proteins from HEK293T cells (Fig. S4). We first evaluated the effect of tRNA on the wild-type MEK2 activity using myelin basic protein (MBP) and ERK2 as substrates (Fig. 4A,B). As a control, we used the same amount of total human RNA which is primarily composed of ribosomal RNAs. Human tRNA decreased the catalytic activity of the wild-type MEK2 by ~2-fold, an effect large enough to merit biological effects. We also tested the inhibitory effect of U0126 on the wild-type MEK2 protein (Fig. S5). Consistent with the *in vivo* results (Fig. 2), U0126 also inhibited the MEK2 activity in the absence and presence of tRNA.

We evaluated the effect of tRNA on the catalytic activity of the various MEK2 mutants using myelin basic protein derived peptide (Fig. 4C,D). tRNA again decreased the activity of the wild-type MEK2 by ~2-fold as well as the S154F mutant and had minimal effects on the activity of the E207K mutant. In contrast, tRNA increased the catalytic activity of the P128Q and Q60P MEK2 mutants. These results indicate that tRNA can affect MEK2 activity differently among the MEK2 mutants.

### Functional consequences of P128Q MEK2 mutation

**Figure 5:**
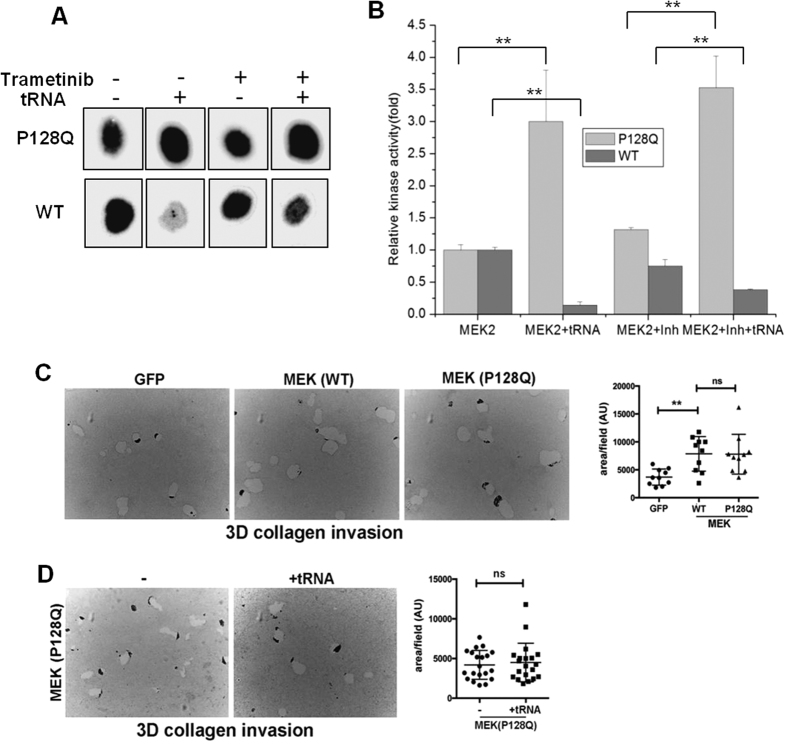
Functional consequences of P128Q MEK2 mutation. (**A**) P128Q MEK2 mutant was affinity purified for *in vitro* kinase assay with or without tRNA in the presence or absence of MEK2 inhibitor trametinib (inh). Wild type MEK2 was included as controls. (**B**) MEK2 kinase activity was quantified, and normalized relative to the kinase activity in the absence of tRNA and trametinib inhibitor. Kinase assays were performed in quadruplicate, and error bars represent standard deviations. **indicates P < 0.05 relative to kinase activity without RNA. (**C**) CD18 cells were transfected with the plasmids of GFP, wild-type MEK, and MEK (P128Q) mutant. The cells were then grown in 3D collagen CD18 cells and treated with EGF (20 ng/ml) for 3 days to induce invasion. The collagen gels were fixed in formalin, embedded in paraffin and sectioned. The collagenolytic paths generated by invading cells were quantified and relative invasion in 3D collagen was determined. **indicates P < 0.05 relative to WT MEK2, ns indicates no significant difference. (**D**) CD18 cells were transfected with 0.5 μg plasmid of MEK (P128Q) and co-transfected with tRNA (1.25 μg). The cells were then grown in 3D collagen, treated with EGF and the relative invasion in 3D collagen was analyzed. The results are representative of three independent experiments. The y-axis of the graphs in (**C**,**D**) are normalized to each parallel performed experimental series (A. U. = arbitrary units).

The P128Q mutant is unique among the MEK2 mutants we studied here. Compared to the wild-type MEK2, it has significantly higher basal activity as well as an enhanced tRNA effects on its activity. We evaluated the functional consequence of the P128Q mutation using both *in vitro* and *in vivo* assays. The MEK inhibitor trametinib can block the catalytic activity of the wild-type MEK2 protein^15^,^16^. In contrast, trametinib failed to block the catalytic activity of the MEK2 (P128Q) mutant, even though it inhibited the activity of the wild-type MEK2 (Fig. 5A,B). Trametinib also failed to inhibit tRNA-mediated increase in the MEK2 (P128Q) catalytic activity. These results indicate that in contrast to the wild-type MEK2, the P128Q mutation not only increased catalytic activity in the absence and in the presence of tRNA, but it is also insensitive to certain MEK inhibitors, and therefore may potentially mediate drug resistance.

We also evaluated the effects of the P128Q mutation using an *in vivo* assay, focusing on 3D collagen invasion by MT1-MMP-expressing CD18 (CD18-MT) cells. MT1-MMP is a key collagenase that has been shown to be important for invasion in 3D collagen^17^,^18^. CD18-MT cells were transfected with GFP, wild-type MEK2, and MEK2 (P128Q) mutant plasmids, grown in 3D collagen, and the collagenolytic paths generated by invading cells were quantified (Fig. 5C). Both wild-type MEK2 and MEK2 (P128Q) mutant increased invasion of CD18-MT cells in 3D collagen.

To examine the effect of tRNA, we directly transfected purified human tRNA into CD18 cells, since it is exceedingly difficult to overexpress tRNA in mammalian cells^19^. In contrast to the tRNA-mediated increase of the MEK2 (P128Q) catalytic activity *in vitro*, direct transfection of human tRNA did not increase 3D invasion by MEK2 (P128Q) (Fig. 5D). Neither, direct transfection of human tRNA did not increase 3D invasion by the wild type MEK2 (Fig. S6). The difference in the *in vitro* and *in vivo* effects of tRNA on MEK2 (P128Q) activity can be due to the fact that there is already a large amount of endogenous tRNA *in vivo*, and that tRNA transfection would not noticeably increase the intracellular tRNA concentration, a common observation in breast cancer cell studies^19^. MEK is localized to the cytoplasm, but a lot of the transfected tRNAs tend to localize in the nucleus^20^. Therefore, transfected tRNA and MEK2 can end up in different intracellular compartments, consistent with the lack of *in vivo* effect of tRNA transfection on MEK2 (P128Q)-driven invasion.

Overall, in this report we show that tRNA and MEK2 can interact in pancreatic cancer cells and this interaction has the potential to alter the catalytic activity of the MEK2 protein. The MEK pathway functions downstream of Kras, which is mutated in >95% of human pancreatic ductal adenocarcinoma (PDAC) tumors, and contributes to the effects of mutant Kras on pancreatic cancer development and progression^21^,^22^,^23^. While the role of MEK1/2 in PDAC development and progression is well established^23^,^24^,^25^, less is known about the effects of MEK2 mutation in pancreatic cancer progression. With the use of MEK inhibitors in pancreatic cancer therapy^26^,^27^, it is likely that cancer cells will acquire MEK mutations. While there is little study about how MEK2 mutants affect the structure of MEK2, it has been proposed that mutations at different positions on MEK2 protein could affect MEK2 functions^28^. Presumably, mutations on MEK2 protein could lead to structural changes and charging level at the tRNA-interacting interface, resulting in different interaction patterns with tRNA. Our results suggest that depending on the mutation, MEK2 interactions with tRNAs may contribute to cancer progression by selectively altering the catalytic activity of the MEK2 protein. These findings increase our understanding of the extra-translational roles of tRNA in regulating cellular function.

## Methods

### Antibodies and Reagents

Antibodies against MEK2 (catalog no. sc-524) and EF1α (catalog no. 28578) were purchased from Santa Cruz Biotechnology, and normal rabbit IgG (catalog no. 2729) was from Cell Signaling Technology. Anti-FLAG M2 affinity gel (catalog no. A2220) was purchased from Sigma. Anti-DDK magnetic beads (catalog no. TA150042) were purchased from OriGene Technologies. FLAG peptide (catalog no. F3290) was purchased from Sigma. Lipofectamine 3000 transfection reagent (catalog no. L3000-001) was acquired from Life Technologies. MEK1/2 inhibitor U0126 (catalog no. 9903) was purchased from Cell Signaling Technology, while the MEK1/2 inhibitor trametinib (catalog no. sc-364639) was obtained from Santa Cruz Biotechnology. Dynabeads protein A (catalog no. 10002D) were purchased from Life Technologies. Cell lysis reagent (catalog no. C2978) for mammalian cells was from Sigma. Human total tRNA (catalog no. BH401) was purchased from Bio S&T Inc, or gel purified from HEK293T cells. Human total RNA was isolated from HEK293T cells. Inactive human ERK2 (catalog no. 14-536) was obtained from EMD Millipore. Myelin basic protein (catalog no. 13-104) was purchased from Millipore and myelin basic protein derived peptide (catalog no. sc-3011) was from Santa Cruz Biotechnology. P81 phosphocellulose squares (catalog no. 20-134) were obtained from Whatman.

### CLIP-Chip in tumorigenic and non-tumorigenic pancreatic cells

The interaction between tRNA and MEK2 protein in pancreatic cancer cells was examined using the established and widely used CLIP-Chip method that identifies the interaction between RNA and proteins through covalent bond formation upon UV irradiation^6^. Pancreatic cancer cell lines (CD18, AsPC-1 and Panc-1 cells) and non-tumorigenic pancreatic cancer cell line HPNE were obtained from American Type Culture Collection (ATCC). Cells grown in 15 cm plates were maintained in DMEM containing 10% FBS and antibiotics (100 U/mL penicillin and 100 μg/mL streptomycin). Cells were treated with DMSO (vehicle control) or 10 μM of the MEK1/2 inhibitor U0126 for 4 h followed by UV crosslinking. Cells at 80% confluency were UV irradiated twice with 400 mJ/cm^2^ at 254 nm. Crosslinked cells were lysed followed by immunoprecipitation with Dynabeads protein A beads using 8 μg MEK2 antibody. EF1α antibody and normal IgG were used as positive and negative control, respectively. After immunoprecipitation, RNA-protein complexes were digested with proteinase K and antibody-bound RNAs were recovered from the beads. The RNA was 3′ ^32^P labeled with T4 RNA ligase 1 and analyzed on 10% denaturing PAGE with yeast tRNA^Phe^ as size control. To further analyze MEK2-binding tRNA species, the corresponding tRNA-sized bands were cut out of the gel for tRNA microarray hybridization. tRNA microarray preparation, hybridization, and data analysis were performed according to methods described previously^4^,^10^. Microarrays were performed with at least two replicates for each experiment.

### Site-directed mutagenesis of MEK2 mutants

Four diseased-derived genetic mutations of MEK2 (Q60P, P128Q, S154F, and E207K) were chosen from published literatures^7^,^8^,^9^. The template pCMV6-MEK2-Myc-DDK (NCBI reference sequence: NP_109587.1, Origene) was used to generate MEK2 mutations using a site-directed mutagenesis kit according to the manufacturer’s description (Agilent). Briefly, 50 ng of template DNA with was reacted with 100 ng/ml of primer pairs for PCR as following temperature: 95 °C for 30 sec, 55 °C for 1 min, 68 °C for 6 min and 15 seconds during 12 cycles. Amplicons were digested with *Dpn I* (New England Biolabs) digestion for *E. coli* transformation. Mutations on MEK2 were validated by sequencing and used for further analysis. Oligonucleotide primers for MEK2 mutations are:

Q60P, forward: CCTTTCTCACCCCGAAAAGCCAAGGTC and reverse: GACCTTGGCTTTCGGGGTGAGAAAGG;

S154F, forward: GAACACATGGACGGCGGCTTTCTGGACCAGGTGCTGAAAG and reverse: CTTTCAGCACCTGGTCCAGAAAGCCGCCGTCCATGTGTTC;

P128Q, forward: GAATGCAACTCGCAGTACATCGTGGGC and reverse: GCCCACGATGTACTGCGAGTTGCATTC;

E207K, forward: GTGAACTCTAGAGGGAAGATCAAGCTGTGTG and reverse: CACACAGCTTGATCTTCCCTCTAGAGTTCAC.

### Interactions of tRNA with MEK2 mutants in cells

30 μg flag-tagged DNA encoding wild-type or mutant MEK2 was transfected into HEK293T cells in 15 cm plates using Lipofectamine 3000 (Invitrogen). Empty plasmid flag-pCMV5 was also transfected as the control. Expression level of transfected MEK2 plasmids was checked with Western blot using anti-flag antibody. For CLIP-Chip experiment, cells overexpressing different MEK2 proteins for 48 h were UV crosslinked followed by immunoprecipitation using 50 μl anti-DDK magnetic beads. RNA-protein complex was treated as above to isolate MEK2-binding RNAs. The tRNA-sized bands were cut out of the denaturing PAGE gel for tRNA microarray analysis. Individual MEK2-binding tRNA species was analyzed and heat maps were plotted to show the relative abundance of specific tRNA species.

### Expression and purification of wild type and mutant MEK2 proteins

HEK293T cells were transfected with 30 μg flag-tagged DNA encoding MEK2 using Lipofectamine 3000 and incubated for 48 h. Cells were lysed in mammalian cell lysis reagent including fresh Protease Inhibitor Cocktails. Cell lysates were centrifuged and the supernatant was used for protein purification with anti-FLAG M2 affinity gel as previously described^29^. Individual flag-tagged MEK2 proteins were eluted with FLAG peptide after stringent washes. The FLAG peptide was removed by Amicon filter of 10 kDa. Protein concentration was measured by Bradford method (Bio-Rad) and protein purity was checked by SDS-PAGE with Coomassie staining.

### *In vitro* kinase activity of wild-type and mutant MEK2

Kinase assay of MEK2 protein consisted of phosphorylation of ERK2 substrate by MEK2^12^,^13^,^14^. To demonstrate the effect of tRNA on MEK2 activity, 500 ng of MEK2 protein was pre-incubated with inactive ERK2 (0.4 μg/μl) in kinase buffer (5 mM HEPES, 3 mM β-glycerophosphate, 5 mM MgCl_2_, 2 mM EGTA, 1 mM EDTA, 0.05 mM DTT) in the presence of human tRNA or human total RNA (0.2 μg/μl). The reactions were initiated with the addition of 50 μM ATP, reactions were incubated at 30 °C for 60 min. Activated mixture was then incubated with myelin basic protein or myelin basic protein-derived peptide (0.2 μg/μl), which is the substrate of ERK2. The reactions were further initiated with the addition of 50 μM non-radioactive and [γ-^32^P]ATP, reactions were incubated at 30 °C for another 60 min. The reaction mixture was spotted onto P81 paper followed by stringent washes with 0.75% phosphoric acid. The P81 papers were exposed to phosphorimagering and quantified using the phosphorimager software. To confirm the effect of tRNA on MEK2 activity, we also performed kinase assays by using inactive ERK2 as direct substrate of MEK2. In the case of using MEK2 inhibitor trametinib or U0126^7^,^30^,^31^, wild type and mutant MEK2 (P128Q) were tested for kinase activity in kinase buffer with or without inhibitor (10 μM), in the presence or absence of yeast tRNA (2 μg/μl).

### Three-dimensional collagen invasion assay

To show MEK2-driven cell invasion in pancreatic cells, CD18-MT cells were transfected with GFP, wild type MEK and mutant MEK (P128Q). The cells were then grown in 3D collagen CD18 cells and treated with EGF (20 ng/ml) for 3 days to induce invasion. The collagen gels were fixed in formalin, embedded in paraffin and sectioned in Pathology Core Facility at Northwestern University. The collagenolytic paths generated by invading cells were quantified and relative invasion in 3D collagen was determined as previously described^18^. We did not perform this assay with a translational inhibitor such as cycloheximide since it decreases migration and invasion^32^, thus would confound our ability to evaluate the role of tRNA in regulating invasion in 3D collagen.

### Statistical analysis

Data in all assays are expressed as Mean ± S.D. of independent replicates, and data between groups were compared by Student’s t test using SPSS16.0 software. Error bars represent standard deviation. The *P* value of <0.05 was considered to be a significant difference.

### tRNA microarray layout

The microarray platform has been deposited in the National Center for Biotechnology Information (NCBI) Gene Expression Omnibus (GEO) database under accession number GPL9428.

## Additional Information

**How to cite this article**: Wang, X. *et al.* Interaction of tRNA with MEK2 in pancreatic cancer cells. *Sci. Rep.* **6**, 28260; doi: 10.1038/srep28260 (2016).

## Supplementary Material

Supplementary Information

## References

[b1] Wek S. A., Zhu S. & Wek R. C. The histidyl-tRNA synthetase-related sequence in the eIF-2 alpha protein kinase GCN2 interacts with tRNA and is required for activation in response to starvation for different amino acids. Mol Cell Biol 15, 4497–4506 (1995). 7623840 10.1128/mcb.15.8.4497 PMC230689

[b2] Li M. *et al.* Codon-usage-based inhibition of HIV protein synthesis by human schlafen 11. Nature 491, 125–128 (2012). 23000900 10.1038/nature11433 PMC3705913

[b3] Katibah G. E. *et al.* tRNA binding, structure, and localization of the human interferon-induced protein IFIT5. Mol Cell 49, 743–750 (2013). 23317505 10.1016/j.molcel.2012.12.015 PMC3615435

[b4] Parisien M. *et al.* Discovering RNA-protein interactome by using chemical context profiling of the RNA-protein interface. Cell Rep 3, 1703–1713 (2013). 23665222 10.1016/j.celrep.2013.04.010 PMC3769137

[b5] Ule J., Jensen K., Mele A. & Darnell R. B. CLIP: a method for identifying protein-RNA interaction sites in living cells. Methods 37, 376–386 (2005). 16314267 10.1016/j.ymeth.2005.07.018

[b6] Zhang C. & Darnell R. B. Mapping *in vivo* protein-RNA interactions at single-nucleotide resolution from HITS-CLIP data. Nat Biotechnol 29, 607–614 (2011). 21633356 10.1038/nbt.1873 PMC3400429

[b7] Villanueva J. *et al.* Concurrent MEK2 mutation and BRAF amplification confer resistance to BRAF and MEK inhibitors in melanoma. Cell Rep 4, 1090–1099 (2013). 24055054 10.1016/j.celrep.2013.08.023 PMC3956616

[b8] Rauen K. A. *et al.* Molecular and functional analysis of a novel MEK2 mutation in cardio-facio-cutaneous syndrome: transmission through four generations. Am J Med Genet A 152A, 807–814 (2010). 20358587 10.1002/ajmg.a.33342 PMC4180666

[b9] Nikolaev S. I. *et al.* Exome sequencing identifies recurrent somatic MAP2K1 and MAP2K2 mutations in melanoma. Nat Genet 44, 133–139 (2012). 22197931 10.1038/ng.1026

[b10] Netzer N. *et al.* Innate immune and chemically triggered oxidative stress modifies translational fidelity. Nature 462, 522–526 (2009). 19940929 10.1038/nature08576 PMC2785853

[b11] Duan W. & Wong W. S. Targeting mitogen-activated protein kinases for asthma. Curr Drug Targets 7, 691–698 (2006). 16787171 10.2174/138945006777435353

[b12] Butch E. R. & Guan K. L. Characterization of ERK1 activation site mutants and the effect on recognition by MEK1 and MEK2. J Biol Chem 271, 4230–4235 (1996). 8626767 10.1074/jbc.271.8.4230

[b13] Sato S., Fujita N. & Tsuruo T. Involvement of 3-phosphoinositide-dependent protein kinase-1 in the MEK/MAPK signal transduction pathway. J Biol Chem 279, 33759–33767 (2004). 15175348 10.1074/jbc.M402055200

[b14] Rebocho A. P. & Marais R. ARAF acts as a scaffold to stabilize BRAF:CRAF heterodimers. Oncogene 32, 3207–3212 (2013). 22926515 10.1038/onc.2012.330

[b15] Gilmartin A. G. *et al.* GSK1120212 (JTP-74057) is an inhibitor of MEK activity and activation with favorable pharmacokinetic properties for sustained *in vivo* pathway inhibition. Clin Cancer Res 17, 989–1000 (2011). 21245089 10.1158/1078-0432.CCR-10-2200

[b16] Yamaguchi T., Kakefuda R., Tajima N., Sowa Y. & Sakai T. Antitumor activities of JTP-74057 (GSK1120212), a novel MEK1/2 inhibitor, on colorectal cancer cell lines *in vitro* and *in vivo*. Int J Oncol 39, 23–31 (2011). 21523318 10.3892/ijo.2011.1015

[b17] Shields M. A., Dangi-Garimella S., Krantz S. B., Bentrem D. J. & Munshi H. G. Pancreatic cancer cells respond to type I collagen by inducing snail expression to promote membrane type 1 matrix metalloproteinase-dependent collagen invasion. J Biol Chem 286, 10495–10504 (2011). 21288898 10.1074/jbc.M110.195628 PMC3060503

[b18] Chow C. R. *et al.* Cancer Cell Invasion in Three-dimensional Collagen Is Regulated Differentially by Galpha13 Protein and Discoidin Domain Receptor 1-Par3 Protein Signaling. J Biol Chem 291, 1605–1618 (2016). 26589794 10.1074/jbc.M115.669606 PMC4722444

[b19] Pavon-Eternod M., Gomes S., Rosner M. R. & Pan T. Overexpression of initiator methionine tRNA leads to global reprogramming of tRNA expression and increased proliferation in human epithelial cells. RNA 19, 461–466 (2013). 23431330 10.1261/rna.037507.112 PMC3677255

[b20] Parisien M., Wang X. & Pan T. Diversity of human tRNA genes from the 1000-genomes project. RNA Biol 10, 1853–1867 (2013). 24448271 10.4161/rna.27361 PMC3917988

[b21] Hezel A. F., Kimmelman A. C., Stanger B. Z., Bardeesy N. & Depinho R. A. Genetics and biology of pancreatic ductal adenocarcinoma. Genes Dev 20, 1218–1249 (2006). 16702400 10.1101/gad.1415606

[b22] Ferro R. & Falasca M. Emerging role of the KRAS-PDK1 axis in pancreatic cancer. World J Gastroenterol 20, 10752–10757 (2014). 25152578 10.3748/wjg.v20.i31.10752 PMC4138455

[b23] Collisson E. A. *et al.* A central role for RAF–>MEK–>ERK signaling in the genesis of pancreatic ductal adenocarcinoma. Cancer Discov 2, 685–693 (2012). 22628411 10.1158/2159-8290.CD-11-0347 PMC3425446

[b24] Hayes T. K. *et al.* Long-Term ERK Inhibition in KRAS-Mutant Pancreatic Cancer Is Associated with MYC Degradation and Senescence-like Growth Suppression. Cancer Cell 29, 75–89 (2016). 26725216 10.1016/j.ccell.2015.11.011 PMC4816652

[b25] Kasuga A. *et al.* A phase I/Ib study of trametinib (GSK1120212) alone and in combination with gemcitabine in Japanese patients with advanced solid tumors. Invest New Drugs 33, 1058–1067 (2015). 26259955 10.1007/s10637-015-0270-2

[b26] Bedard P. L. *et al.* A phase Ib dose-escalation study of the oral pan-PI3K inhibitor buparlisib (BKM120) in combination with the oral MEK1/2 inhibitor trametinib (GSK1120212) in patients with selected advanced solid tumors. Clin Cancer Res 21, 730–738 (2015). 25500057 10.1158/1078-0432.CCR-14-1814

[b27] Infante J. R. *et al.* A randomised, double-blind, placebo-controlled trial of trametinib, an oral MEK inhibitor, in combination with gemcitabine for patients with untreated metastatic adenocarcinoma of the pancreas. Eur J Cancer 50, 2072–2081 (2014). 24915778 10.1016/j.ejca.2014.04.024

[b28] Bromberg-White J. L., Andersen N. J. & Duesbery N. S. MEK genomics in development and disease. Brief Funct Genomics 11, 300–310 (2012). 22753777 10.1093/bfgp/els022 PMC3398258

[b29] Wang X. & Pan T. Methionine Mistranslation Bypasses the Restraint of the Genetic Code to Generate Mutant Proteins with Distinct Activities. PLoS Genet 11, e1005745 (2015). 26709516 10.1371/journal.pgen.1005745 PMC4692448

[b30] Caunt C. J., Sale M. J., Smith P. D. & Cook S. J. MEK1 and MEK2 inhibitors and cancer therapy: the long and winding road. Nat Rev Cancer 15, 577–592 (2015). 26399658 10.1038/nrc4000

[b31] Infante J. R. *et al.* Safety, pharmacokinetic, pharmacodynamic, and efficacy data for the oral MEK inhibitor trametinib: a phase 1 dose-escalation trial. Lancet Oncol 13, 773–781 (2012). 22805291 10.1016/S1470-2045(12)70270-X

[b32] Xie Y. *et al.* Breast cancer migration and invasion depend on proteasome degradation of regulator of G-protein signaling 4. Cancer Res 69, 5743–5751 (2009). 19549919 10.1158/0008-5472.CAN-08-3564 PMC2741027

