# Supplementary material for: Interaction of tRNA with MEK2 in pancreatic cancer cells
Source: Sci Rep. 2016 Jun 15;6:28260. doi: 10.1038/srep28260 (PMC4908586; doi:10.1038/srep28260)
Supplement: Supplementary Information [file srep28260-s1.pdf]

Supplementary information

Interaction of tRNA with MEK2 in pancreatic cancer cells

Xiaoyun Wang[1], Christina R. Chow[2], Kazumi Ebine[2], Jiyoung Lee[3], Marsha R. Rosner[3], Tao Pan[1,*], Hidayatullah G Munshi[2,*]

[1] Department of Biochemistry and Molecular Biology, [3]Ben May Department for Cancer Research, University of Chicago, Chicago, IL 60637, USA
[2] Department of Medicine and the Robert H. Lurie Comprehensive Cancer Center, Northwestern University, Chicago, IL 60611, USA

*Corresponding authors:
H.G. Munshi (h-munshi@northwestern.edu), T. Pan (taopan@uchicago.edu).

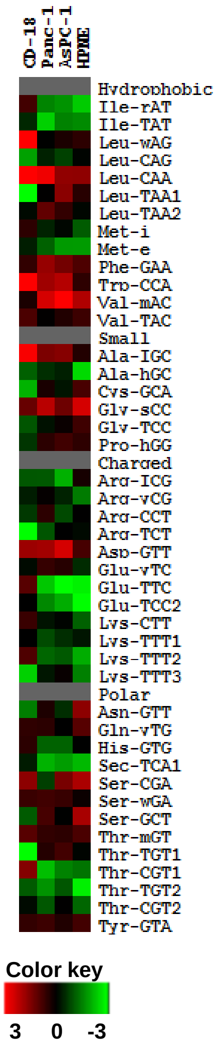

Fig. S1. **Heat map shown as semi-quantitative analysis of specific tRNA in pancreatic cell lines.** Total RNAs were isolated from pancreatic cell lines (CD-18, Panc-1, AsPC-1, HPNE), the same amount of total RNA was 3′ ^{32}P labeled and analyzed on denaturing PAGE with yeast tRNA^{Phe} as size control The labeled tRNA bands were cut out for tRNA microarray hybridization. Anticodon specific tRNA signals are calculated and expressed as $\log_2(X)$, where X indicates values relative to the median value of each array. Relative abundance of specific tRNAs are shown as heat map and are indicated with colors ranging from green (low) to red (high).

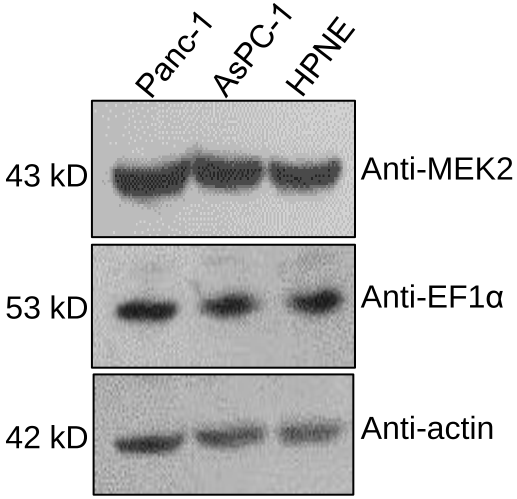

Fig. S2. Western blot of MEK2 expression in pancreatic cells. Expression level of MEK2 and EF1α in three pancreatic cell lines (Panc-1, AsPC-1 and HPNE) was determined using corresponding antibodies, and β-actin was used as loading control.

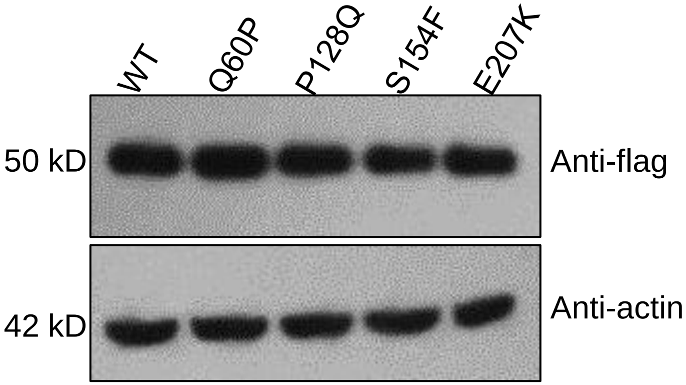

Fig. S3. Transfection and overexpression of MEK2 proteins. Western blots of flag-tagged WT and mutant MEK2 proteins. Expression level of wild type and mutant MEK2 was determined using anti-flag antibody, and β-actin was used as loading control.

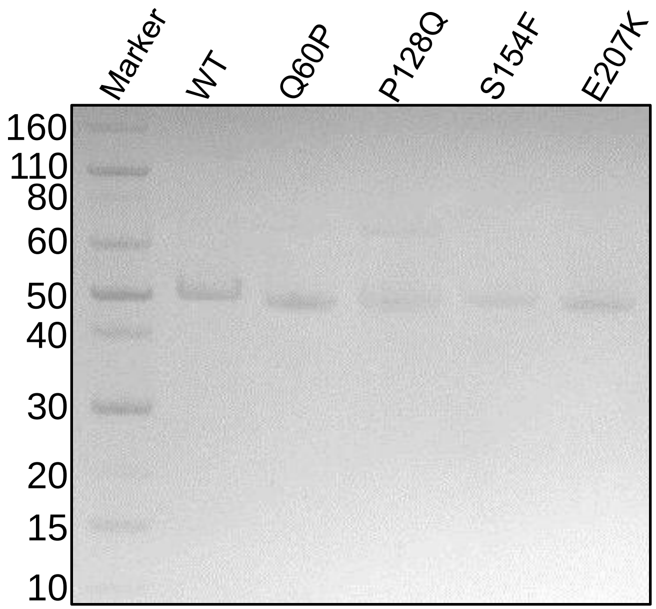

Fig. S4. Affinity purified MEK2 wild-type and mutant proteins from HEK293T cells analyzed by SDS-PAGE with coomassie brilliant blue staining.

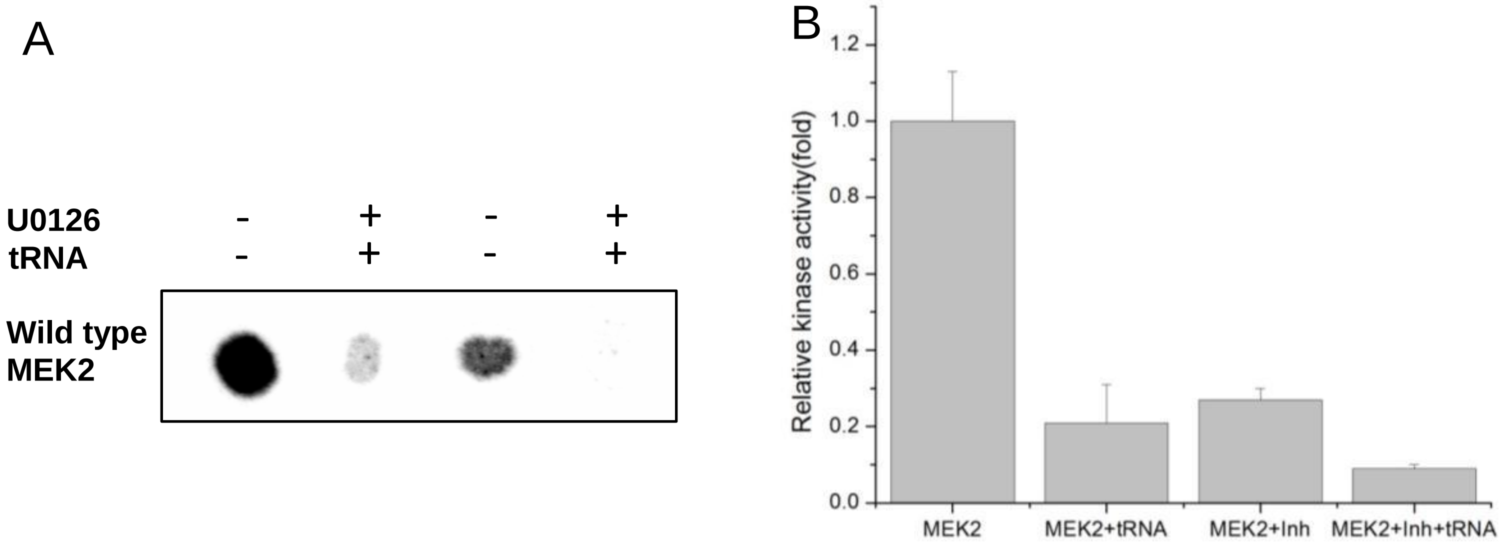

Fig. S5. Wild-type MEK2 kinase assay with and without U0126 inhibitor. (A) Dot blots. (B) MEK2 kinase activity was quantified, and normalized relative to the kinase activity in the absence of tRNA and U0126 inhibitor (inh). Kinase assays were performed in quadruplicate, and error bars represent standard deviations.

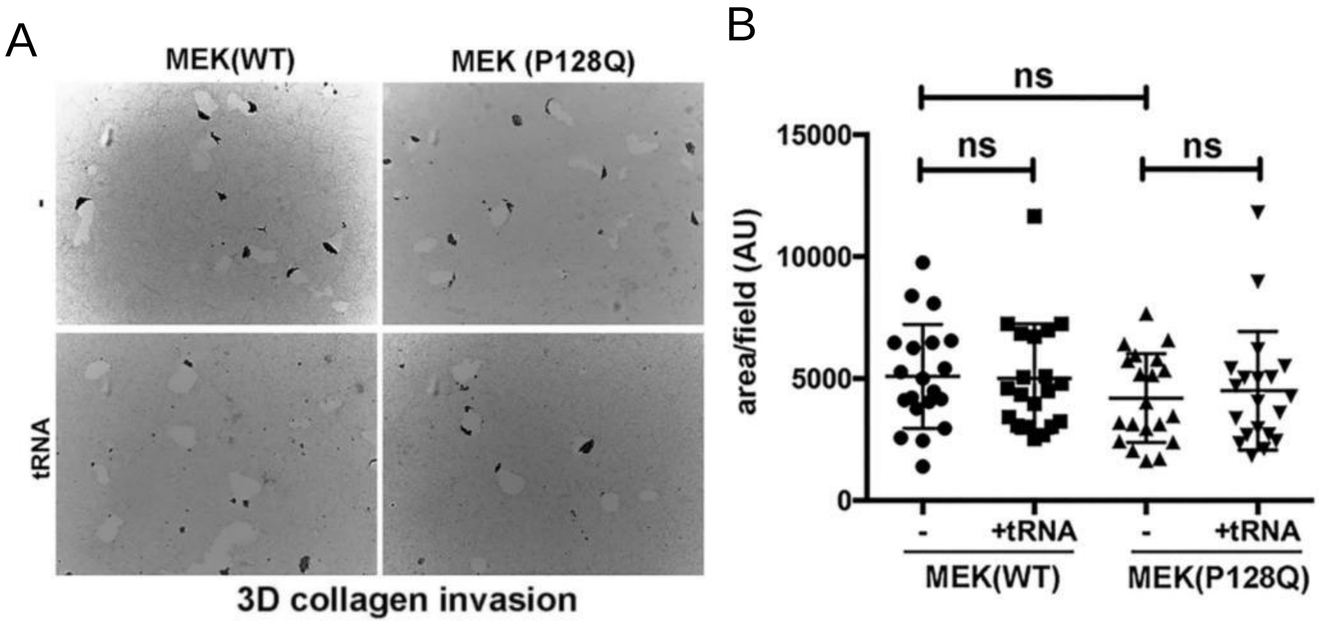

Fig. S6. Comparison of WT and P128Q mutant in 3D collagen invasion. (A) CD18 cells were transfected with 0.5 µg plasmid of wild type MEK2 or P128Q and co-transfected with tRNA (1.25 µg). The cells were then grown in 3D collagen, treated with EGF and the relative invasion in 3D collagen was analyzed. The results are representative of three independent experiments. (B) The collagenolytic paths generated by invading cells were quantified and relative invasion in 3D collagen was determined. ns indicates no significant difference.
